# Supplementary material for: Inhibiting the Ca2+ Influx Induced by Human CSF
Source: Cell Rep. 2017 Dec 12;21(11):3310–6. doi: 10.1016/j.celrep.2017.11.057 (PMC5745229; doi:10.1016/j.celrep.2017.11.057)
Supplement: Document S1. Figures S1–S3 and Tables S1–S3 [file mmc1.pdf]

Cell Reports, Volume *21*

Supplemental Information

Inhibiting the Ca^{2+} Influx Induced by Human CSF

Anna Drews, Suman De, Patrick Flagmeier, David C. Wirthensohn, Wei-Hsin Chen, Daniel R. Whiten, Margarida Rodrigues, Cécile Vincke, Serge Muyldermans, Ross W. Paterson, Catherine F. Slattery, Nick C. Fox, Jonathan M. Schott, Henrik Zetterberg, Christopher M. Dobson, Sonia Gandhi, and David Klenerman

Supplemental Figures:

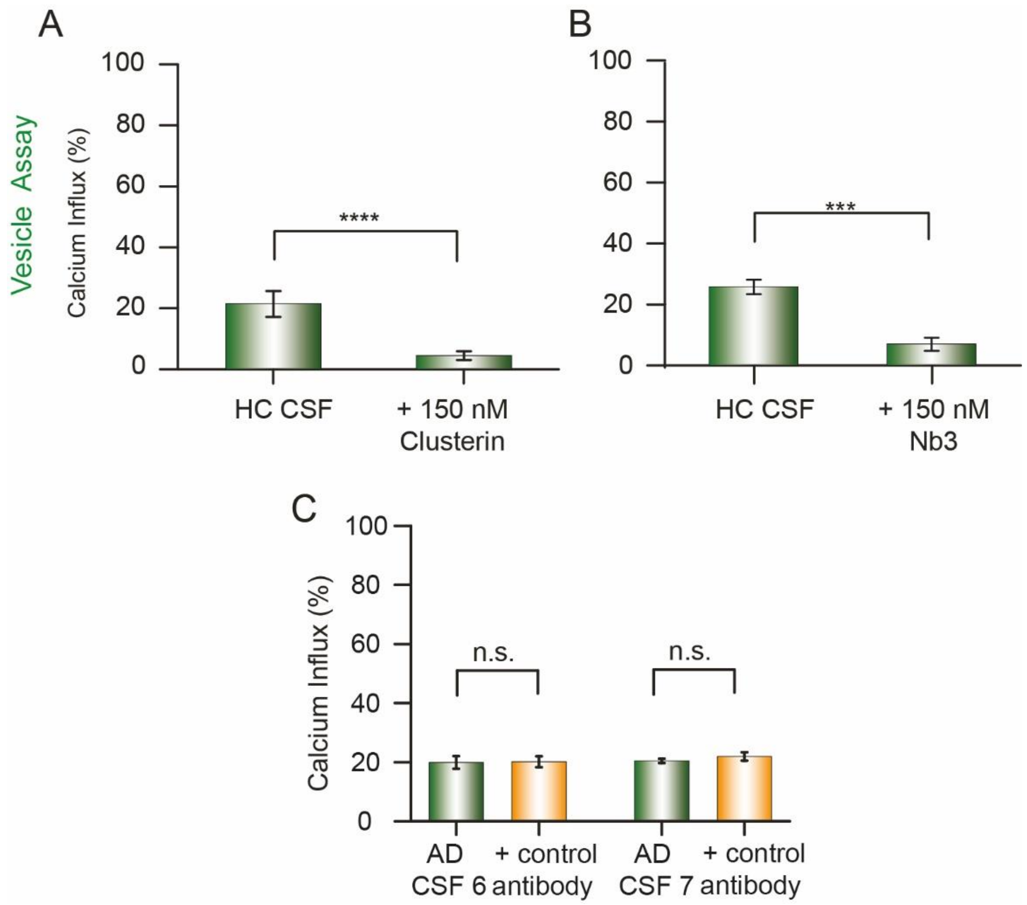

Figure S1. Assessing the ability of Clusterin and Nb3 to counteract the calcium influx caused by human HC CSF and effect of control anti GFP antibody on CSF induced Calcium influx. Related to Figure 2. Inhibition of the calcium influx caused by HC CSF with Clusterin **(A)** and Nb3 **(B)** ,the error bars represent SEM. The statistical data for the experiments are summarized in Table S1. **(C)** A control antibody, 1 μM anti GFP antibody, does not show any significant change in CSF induced Calcium influx.

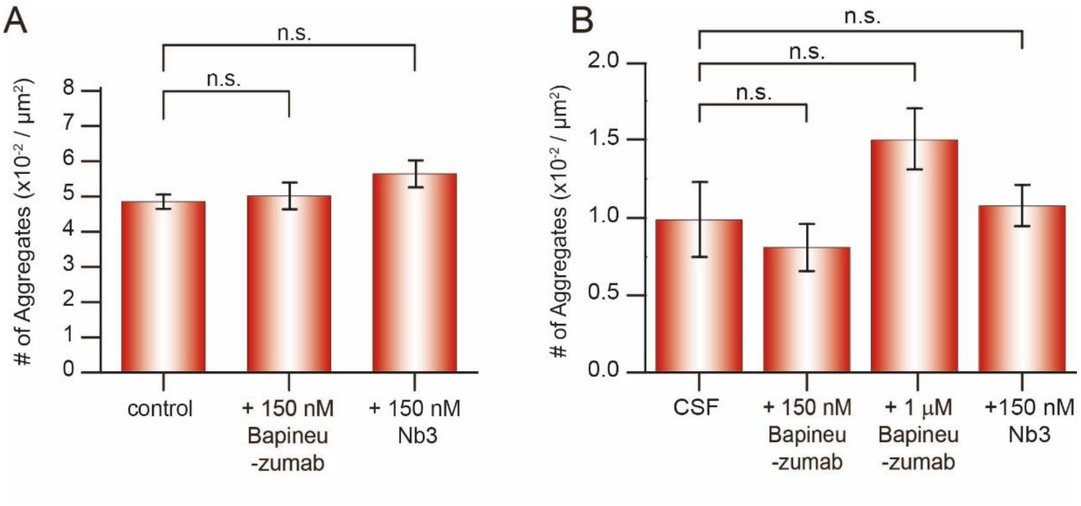

Figure S2. Effect of Antibodies on Thioflavin T positive Aβ42 aggregates and human CSF. **Related to Figure 3. (A)** Aβ42 was aggregated at 5 µM for 5h at 37° C in PBS. An aggregated solution of 1 µM total Aβ4*2* monomer concentration was incubated with 150 nM of each antibody for 15 min and aggregates were counted as described previously. Each bar represents the mean of 3 slides with 27 field of views overall imaging a 3 x 10^6 μm^2 area. Error bars represent the standard error of the mean of all 27 field of views **(B)** The effect of the antibodies on the number of ThT positive aggregates in human CSF. The mean of 4 samples with 27 field of views each with the error bars representing the standard error of the mean of all samples. Full statistical details are found in Table S3.

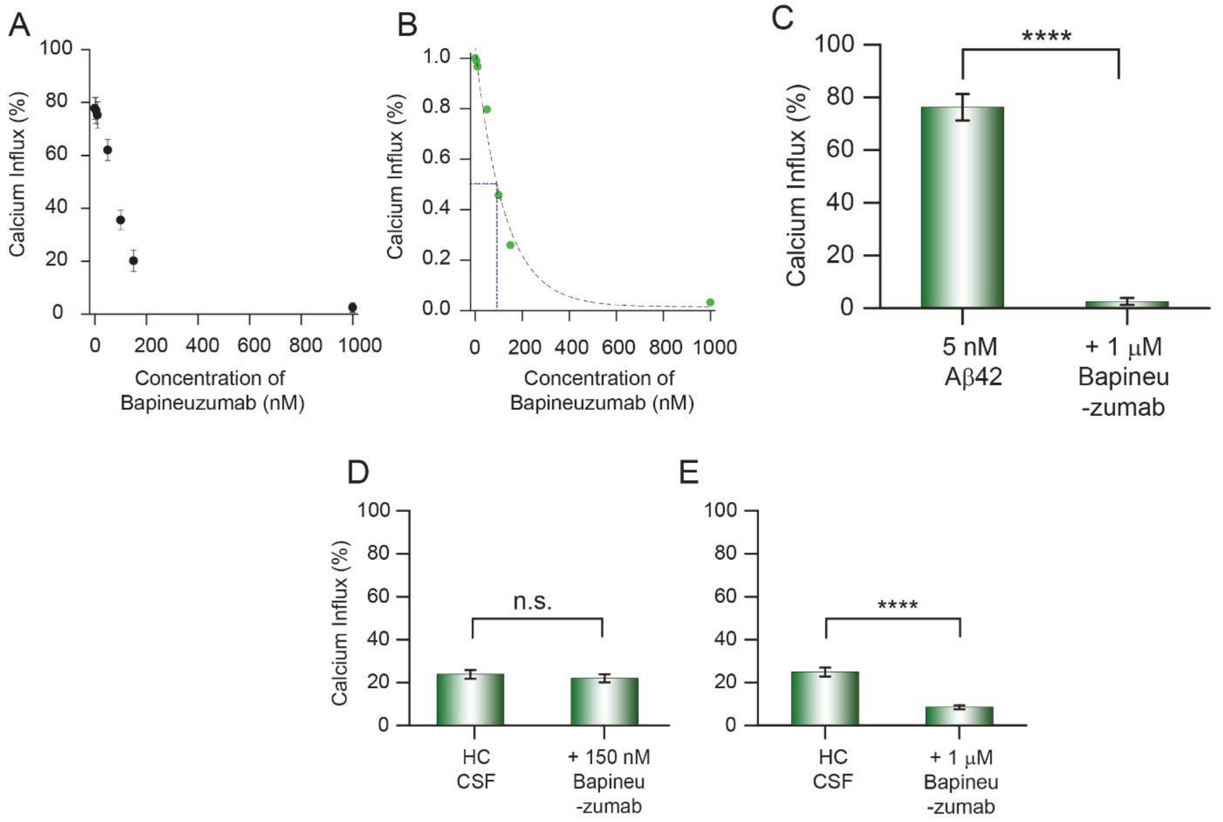

Figure S3. Inhibition of CSF induced Calcium influx by Bapineuzumab. Related to Figure 3. Recombinant Aβ42 was incubated at a concentration of 2 μM at 37 °C and an aliquot was taken from the reaction at a time point corresponding to the end of the lag-phase [1]. **(A)** The aliquot was diluted to a concentration of 5 nM and incubated with increasing concentrations of Bapineuzumab. **(B)** The Ca^{2+} influx normalized to the influx in the absence of Bapineuzumab. The Ca^{2+} influx caused by the Aβ42 aggregates was reduced by half at a concentration of approximately 90 nM of Bapineuzumab (indicated by the blue line). Lines are guides to the eye. **(C)** Inhibition of the calcium influx caused by recombinant Aβ42 aggregates by the antibody Bapineuzumab at a concentration of 1 μM measured with the vesicle assay. Error bars are SEM. **(D)** and **(E)** Inhibition of the calcium influx caused by human HC CSF into vesicles by 150 nM and 1μM Bapineuzumab respectively. The error bars are SEM.

Supplemental Tables:

Table S1: Statistical data to all vesicle assays Related to Figure 1, 2 and 3.

Single Vesicle Assay				
**Figure No.**	**Sample**	**Total vesicle analysed**	**No of CSF samples**	**P Value**
**1D**	***AD***	4906	5	0.440500
	HC	4602	5	
**2A**	***AD***	5032	5	0.000033
	AD+ Clusterin	4876	5	
**Fig. S1A**	***HC***	4905	5	0.000040
	HC+ Clusterin	5536	5	
**2B**	***AD***	4690	5	0.000016
	AD+ Nb3	4883	5	
**Fig. S1B**	***HC***	4925	5	0.000110
	HC + Nb3	4754	5	
**3A**	***Aβ42***	5826	5	0.000034
	Aβ42+ 150nM BAPI	4869	5	
**3B**	***AD***	5429	5	0.18411
	AD+ 150 nM BAPI	5202	5	
**3C**	***AD***	5277	5	0.00112
	AD+ 1 µm BAPI	5509	5	

Table S2: Statistical data to all cell assays. Related to Figure 1, 2 and 3.

Cell Assay				
**Figure No.**	**Sample**	**No of Cells used**	**No of CSF samples**	**P Value**
**1E**	***AD***	33	6	0.306118
	HC	65	9	
**2C**	***AD***	25	4	0.001751
	AD+ Clusterin	19	4	
**2D**	***AD***	33	4	0.000591
	AD+ Nb3	24	4	
**3D**	***Aβ42***	8	1	0.004945
	Aβ42 + BAPI	6	1	
**3E**	***AD***	37	4	0.279328
	AD + 150 nM BAPI	25	4	
**3F**	***AD***	31	5	0.166927
	AD + 1000 nM BAPI	27	5	

Table S3: Statistical data to all SAVE measurements. Related to Figure 1.

Single Aggregate Measurements				
**Figure No.**	**Sample**	**Areas imaged per sample (overall 3 x 10^6 μm^2)**	**No of CSF samples**	**P Value**
**1F**	***AD***	27	19	0.4907
	HC	27	12	
**S3A**	***Aβ42***	27	N/A	0.703221
	Aβ42 + BAPI	27	N/A	
**S3A**	***Aβ42***	27	N/A	2.05552
	Aβ42 + Nb3	27	N/A	
**S3B**	***CSF***	27	4	0.626095
	CSF + 150 nM BAPI	27	4	
**S3B**	***CSF***	27	4	0.289404
	CSF + 1000 nM BAPI	27	4	
**S3B**	***CSF***	27	4	0.730615
	CSF + 150 nM Nb3	27	4	
