# Supplementary material for: Inhibiting the Ca2+ Influx Induced by Human CSF
Source: Cell Rep. 2017 Dec 12;21(11):3310–6. doi: 10.1016/j.celrep.2017.11.057 (PMC5745229; doi:10.1016/j.celrep.2017.11.057)
Supplement: Document S2. Article plus Supplemental Information [file mmc2.pdf]

Resource

Cell Reports

Inhibiting the Ca^{2+} Influx Induced by Human CSF

Graphical Abstract

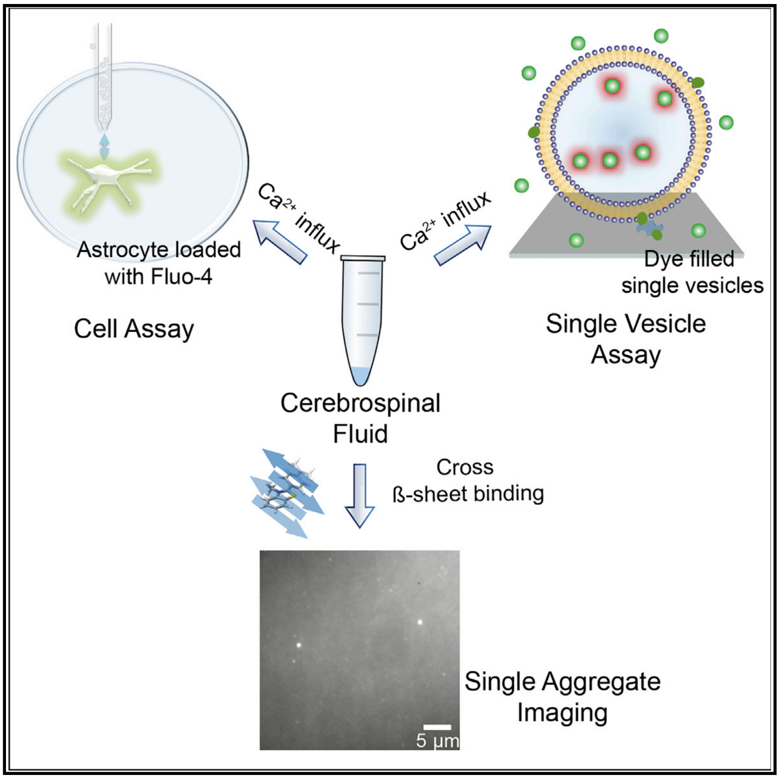

Highlights

- Human cerebrospinal fluid (CSF) can permeabilize membranes and induce Ca^{2+} influx
- CSF of control individuals and those with Alzheimer's disease show similar Ca^{2+} influx
- An extracellular chaperone clusterin and a nanobody Nb3 can inhibit Ca^{2+} influx
- Bapineuzumab reduces Aβ-aggregate-induced Ca^{2+} influx but is less effective in CSF

Authors

Anna Drews, Suman De, Patrick Flagmeier, ..., Christopher M. Dobson, Sonia Gandhi, David Klenerman

Correspondence

dk10012@cam.ac.uk

In Brief

Drews et al. develop and implement sensitive *in vitro* assays to quantitatively measure the Ca^{2+} influx caused by human cerebrospinal fluid. If a given chaperone, antibody, or nanobody is effective in reducing Ca^{2+} influx, the authors determine what concentration is needed to prevent Ca^{2+} influx.

Drews et al., 2017, Cell Reports *21*, 3310–3316
December 12, 2017 © 2017 The Authors.
https://doi.org/10.1016/j.celrep.2017.11.057

CellPress

OPEN ACCESS
CellPress

Cell Reports
Resource

Inhibiting the Ca^{2+} Influx Induced by Human CSF

Anna Drews,[1,7] Suman De,[1,7] Patrick Flagmeier,[1,7] David C. Wirthensohn,[1,7] Wei-Hsin Chen,[1] Daniel R. Whiten,[1] Margarida Rodrigues,[1] Cécile Vincke,[2] Serge Muyldermans,[2] Ross W. Paterson,[3] Catherine F. Slattery,[3] Nick C. Fox,[3] Jonathan M. Schott,[3] Henrik Zetterberg,[4,5] Christopher M. Dobson,[1] Sonia Gandhi,[5] and David Klenerman[1,6,8,*]
[1]Department of Chemistry, University of Cambridge, Lensfield Road, Cambridge CB2 1EW, UK
[2]Laboratory of Cellular and Molecular Immunology, Vrije Universiteit Brussel, Brussels, Belgium
[3]Dementia Research Centre, UCL Institute of Neurology, Queen Square, London WC1N 3BG, UK
[4]Clinical Neurochemistry Laboratory, Department of Psychiatry and Neurochemistry, Institute of Neuroscience and Physiology, the Sahlgrenska Academy, University of Gothenburg, Mölndal, Sweden
[5]Sobell Department of Motor Neuroscience and Movement Disorders, UCL Institute of Neurology, Queen Square, London WC1N 3BG, UK
[6]UK Dementia Research Institute, University of Cambridge, Cambridge CB2 0XY, UK
[7]These authors contributed equally
[8]Lead Contact
*Correspondence: dk10012@cam.ac.uk
https://doi.org/10.1016/j.celrep.2017.11.057

SUMMARY

One potential therapeutic strategy for Alzheimer's disease (AD) is to use antibodies that bind to small soluble protein aggregates to reduce their toxic effects. However, these therapies are rarely tested in human CSF before clinical trials because of the lack of sensitive methods that enable the measurement of aggregate-induced toxicity at low concentrations. We have developed highly sensitive single vesicle and single-cell-based assays that detect the Ca^{2+} influx caused by the CSF of individuals affected with AD and healthy controls, and we have found comparable effects for both types of samples. We also show that an extracellular chaperone clusterin; a nanobody specific to the amyloid-β peptide (Aβ); and bapineuzumab, a humanized monoclonal antibody raised against Aβ, could all reduce the Ca^{2+} influx caused by synthetic Aβ oligomers but are less effective in CSF. These assays could be used to characterize potential therapeutic agents in CSF before clinical trials.

INTRODUCTION

Protein misfolding and aggregation underlies a range of neurodegenerative diseases, including Alzheimer's disease (AD). Monomeric amyloid-β peptide (Aβ) is released from the amyloid precursor protein (APP) by proteolysis, aggregates into a range of soluble oligomers, and, ultimately, aggregates into insoluble amyloid fibrils deposited in the brain (Chiti and Dobson, 2017; Glenner and Wong, 1984; Riek and Eisenberg, 2016). A body of evidence indicates that Aβ oligomers play a key role in the development of AD by initiating a cascade of biochemical processes (including disruption of Ca^{2+} homeostasis) that ultimately lead to neuronal death (Arbel-Ornath et al., 2017; Benilova et al., 2012; Chiti and Dobson, 2017; Haass and Selkoe, 2007). Picomolar concentrations of synthetic Aβ oligomers can cause Ca^{2+} influx into neuronal cells and astrocytes, leading to oxidative stress and caspase 3 activation, which can result in apoptosis (Narayan et al., 2014). The soluble aggregates present within human biofluids have not been studied in such detail, and indeed Aβ can be post-translationally modified and has the potential to interact with other proteins present in the brain (Luo et al., 2016). Moreover, it is unclear which of the aggregates present are most neurotoxic and most directly contribute to the onset of disease. We have in the past lacked the techniques needed to measure and quantify effectively such species present *in vivo*, for example, in human cerebrospinal fluid (CSF), where the concentrations are approximately 1 pM (Savage et al., 2014; Yang et al., 2015). This is an important problem since a number of therapeutic strategies are based on the use of antibodies or nanobodies to reduce the number of or the toxic effects of protein aggregates.

Previous work to test potential therapeutic strategies, for example antibodies, has used aggregates derived from human sources (Walsh et al., 2002), such as those isolated from the soluble fraction of brain homogenates (Shankar et al., 2008). However, this method involves several preparative steps and may lead to soluble aggregates being formed by dissociation from any insoluble fibrils present. A small number of experiments have, however, been previously performed using human CSF without requiring any preparative steps. CSF freely exchanges with the interstitial fluid in the brain, making it a medium that is both available for analysis and contains aggregates formed within a living individual. These experiments have shown that the aggregates present in CSF can induce long-term potentiation deficit in brain slices which can be suppressed by the addition of antibodies raised against Aβ (Klyubin et al., 2008; Walsh et al., 2002). CSF from individuals diagnosed with AD has also been shown to cause cell death, which can be reduced significantly by the addition of physiological amounts of extracellular chaperones (Yerbury and Wilson, 2010).

RESULTS

We have previously developed an extremely sensitive and high-throughput assay based on the quantitative measurement of

 © 2017 The Authors.
This is an open access article under the CC BY license (http://creativecommons.org/licenses/by/4.0/).

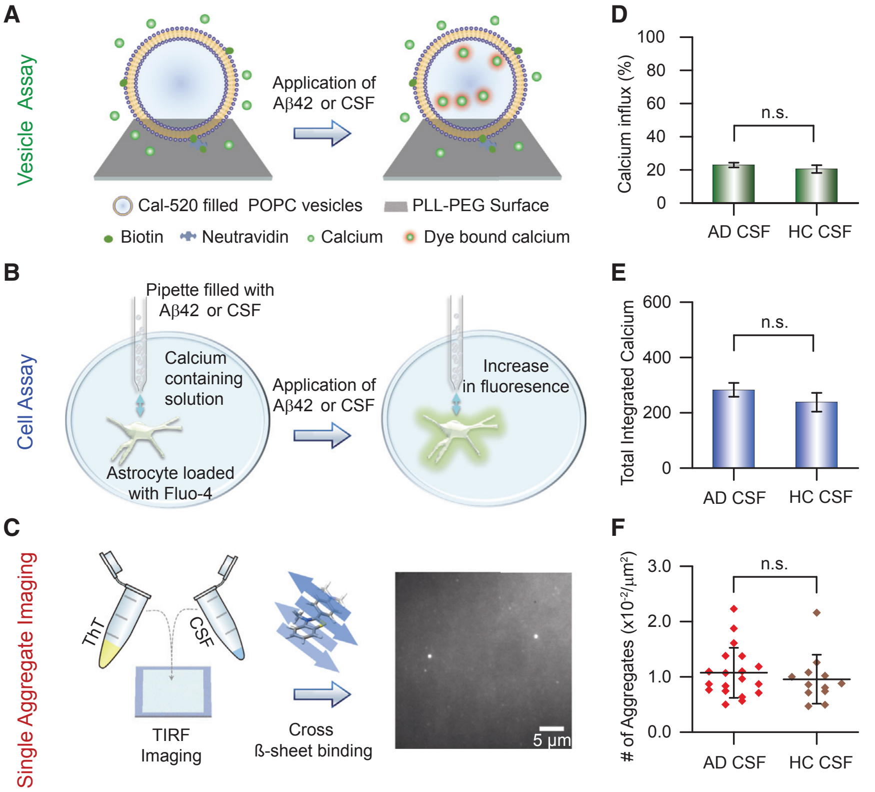

Figure 1. Biophysical Methods Used Here to Study Properties of Human CSF

(A) Quantitative ultrasensitive single vesicle assay to assess the ability of species (e.g., Aβ42 aggregates or the species present in human CSF) to permeate the vesicle membrane. Hundreds of vesicles filled with the Ca^{2+} sensitive dye Cal-520 act as optochemical probes and are tethered to a PLL-PEG-coated glass coverslip using biotin-neutrAvidin linkage. The addition of species able to penetrate the membrane and hence enable Ca^{2+} influx from the surrounding solution results in a change of the fluorescence intensity that can be measured using total internal reflection microscopy (TIRFM) (see Experimental Procedures for details).

(B) Scanning ion conductance microscopy approach to assess the ability of samples (e.g., Aβ42 aggregates or human CSF) to permeate the cell membrane. Upon local delivery of the sample by a nanopipette to the surface of an individual astrocyte loaded with the Ca^{2+} sensitive dye Fluo-4, a change in fluorescence intensity can be detected as a result of the permeation of the cell membrane.

(C) Single aggregate visualization through enhancement (SAVE) imaging using the dye ThT to measure the number of cross β-sheet containing species in a sample (e.g. human CSF). The sample is added on a passivated glass slide, and the number of fluorescent species is measured using TIRFM.

(D) Comparison of the influx of Ca^{2+} ions into vesicles caused by CSF samples from healthy individuals (HC CSF) and individuals affected with AD (AD CSF). The difference between Ca^{2+} influx-induced AD CSF and HC CSF are not significant. Error bars, SEM.

(E) Comparison of the Ca^{2+} influx into astrocytes caused by HC CSF and AD CSF. Error bars, SEM.

(F) Comparison of the number of β-sheet containing species in HC CSF and AD CSF. Error bars, the 25th and the 75th percentiles. Full statistics for all experiments are summarized in Tables S1, S2, and S3.

oligomer-induced Ca^{2+} influx into hundreds of surface immobilized single lipid vesicles (Flagmeier et al., 2017) (Figure 1A). We have also shown that even low levels of Aβ42 oligomers can induce Ca^{2+} influx into neuronal cells by local dosing with a nanopipette (Figure 1B) (Drews et al., 2016). Local dosing reduces the diffusion time to the cell surface, leading to detectable Ca^{2+} influx at low picomolar concentrations of oligomers that is comparable to the concentration of species present in CSF. We have also recently developed a Thioflavin-T (ThT)-based imaging method to count the total number of ThT-active species in CSF using sensitive single-molecule fluorescence measurements (Horrocks et al., 2016) (Figure 1C). Herein we describe how these methods have been used in a series of experiments to characterize the protein aggregates present in the CSF of individuals affected with AD (AD CSF) and control individuals (HC CSF). We have also tested the effectiveness of a single-chain nanobody raised against the Aβ peptide, the extracellular chaperone clusterin, and bapineuzumab (a humanized monoclonal antibody that was recently used in an unsuccessful AD drug trial) (Blennow et al., 2012; Liu et al., 2015; Salloway et al., 2014; Vandenberghe et al., 2016) at reducing the Ca^{2+} influx induced by samples of CSF.

We first carried out experiments using the high-throughput vesicle assay or the cell assay to measure the extent of Ca^{2+} influx induced by samples of AD CSF and HC CSF. We detected no difference in the extent of Ca^{2+} influx between these two sets of samples (Figures 1D and 1E; Tables S1 and S2). A separate set of experiments also showed no significant difference in the number of ThT-active species detected in AD CSF and in HC CSF (Figure 1F; Table S3). Previous work using ELISA-based detection of the total number of aggregates in CSF also showed no clear difference between AD and HC samples (Savage et al., 2014; Yang et al., 2015), a result consistent with our findings. Together, these data suggest that comparable numbers of species in AD CSF and in HC CSF cause Ca^{2+} influx.

To identify which species present in the CSF might be responsible for the Ca^{2+} influx, we added clusterin to AD CSF and to HC CSF (Figures 2 and S1). Clusterin is an extracellular chaperone known to bind misfolded and aggregated proteins at sub-stoichiometric concentrations compared to the monomer concentration (Yerbury et al., 2007). Additionally, multiple clusterin molecules were found to bind to each Aβ oligomer (Narayan et al., 2011, 2012). Moreover, in previous work, we determined that 100 pM clusterin halved the Ca^{2+} influx induced by recombinant Aβ42 oligomers (Flagmeier et al., 2017). This result shows that clusterin has a very high affinity for oligomers of Aβ and, in our experiments with CSF, we used clusterin concentrations more than 100 times greater than 100 pM. Clusterin was very effective at suppressing the Ca^{2+} influx induced by CSF as measured using the vesicle assay (Figures 2A and S1A) and cell

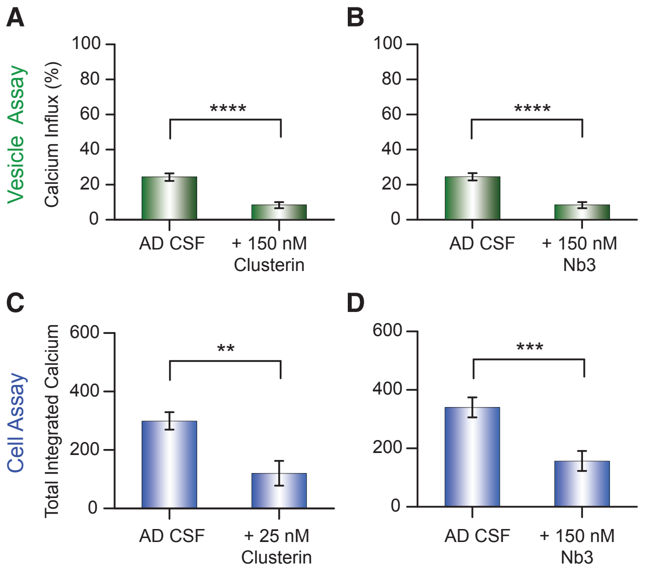

Figure 2. Assessing the Ability of Chaperones and Nanobodies to Counteract the Influx of Ca^{2+} Ions Resulting from the Addition of Aliquots of Human CSF

(A and B) Inhibition of the influx of Ca^{2+} ions into individual vesicles caused by AD CSF by clusterin (150 nM) (A) and the nanobody Nb3 (150 nM) (B).
(C and D) Inhibition of the influx of Ca^{2+} ions into astrocytes caused by human CSF of individuals suffering from AD by clusterin (25 nM) (C) and the nanobody Nb3 (150 nM) (D). The statistical data for the experiments are summarized in Tables S1 and S2. Error bars, SEM.

assay (Figure 2C). That clusterin prevents this Ca^{2+} influx in both assays suggests that protein aggregates present in CSF are responsible for the Ca^{2+} influx. Furthermore, since clusterin is already present in CSF at a concentration of about 90 nM both for individuals affected with AD and for HC (Vranová et al., 2014), this result suggests that in CSF such concentrations are not high enough to be totally effective at reducing Ca^{2+} influx. This finding is in agreement with a previous report that the addition of higher concentrations of clusterin could reduce the toxic effects on cells induced by CSF (Yerbury and Wilson, 2010).

We then tested the effectiveness of a single-chain nanobody Nb3, raised against the Aβ peptide, at reducing CSF-induced Ca^{2+} influx. Nb3 binds to the epitope 17–28 of the Aβ peptide with a measured dissociation constant for the monomeric protein of 13 nM. We have previously shown using the vesicle assay that Nb3 reduces the Ca^{2+} influx induced by recombinant Aβ42 oligomers in the absence of CSF and that approximately 18 nM Nb3 was needed to halve the influx (Flagmeier et al., 2017). We also found that the addition of Nb3 to Aβ42 oligomers significantly reduced the Ca^{2+} influx in the cell assays (Drews et al., 2016). In these experiments, we pre-incubated the AD or HC CSFs with 150 nM Nb3 for 15 min and then performed experiments using our vesicle and cell assays (Figures 2B, 2D, and S1B). These experiments show that Nb3 is able to reduce the extent of Ca^{2+} influx induced by samples of AD CSF and HC CSF. In a control experiment we showed that the addition of a GFP antibody caused no significant reduction in Ca^{2+} influx (Figure S1C). Moreover, the addition of the Nb3 nanobody did not detectably alter the number of ThT-active species (Figure S2), indicating that the addition of the nanobody does not lead to the disaggregation of the aggregates within the timescale of our experiments, approximately 15 min. The observation that Nb3 can reduce the Ca^{2+} influx caused by Aβ oligomers and also by CSF both in the vesicle and in the cellular assays suggests that at least a portion of the influx-inducing species present in CSF contains the amino acids 17–28 of the Aβ sequence. Presumably, Nb3 binds to the species that are potent at inducing Ca^{2+} influx and thereby reduces or prevents their interaction with the membranes of vesicles or cells.

Next, we probed the efficacy of bapineuzumab, which is a bivalent antibody that binds soluble Aβ monomer and oligomers at the N terminus (Miles et al., 2013). Using recombinant Aβ42 oligomers, we found that ~90 nM bapineuzumab was required to halve the influx of Ca^{2+} ions in the vesicle assay (Figures S3A and S3B). Using the vesicle assay we found that 150 nM bapineuzumab caused a relative decrease of 70% in the Ca^{2+} influx (Figure 3A), and that 1 μM bapineuzumab reduced the Ca^{2+} influx completely (Figure S3C). Bapineuzumab also prevented Ca^{2+} influx in the cell assay (Figures 3B and 3D). We then tested 150 nM bapineuzumab on samples of AD CSF and of HC CSF. Addition of the antibody did not detectably alter the number of ThT-active species (Figure S2), indicating that the addition of bapineuzumab does not significantly alter the number of β-sheet containing species within the timescale of our experiments (Table S3). In the vesicle assay, using an antibody at a concentration of 150 nM, we observed an insignificant relative decrease in the Ca^{2+} influx induced by AD CSF and HC CSF of ~12% (Figures 3B and S3D). A 10-fold higher concentration of bapineuzumab, 1 μM, caused a relative decrease in Ca^{2+} influx of ~75% (absolute decrease 18%) (Figures 3C and S3E). However, we observed no reduction in Ca^{2+} influx in the cellular assay at either antibody concentration (Figures 3E and 3F). In the cell assay, this higher antibody concentration is presumably still insufficient to prevent the interaction with the cell membrane, possibly because the aggregates have a higher affinity for the cell membrane than for the membrane of a vesicle. Overall, the results of the single vesicle experiments show that higher bapineuzumab concentrations are needed to reduce the extent of Ca^{2+} influx caused by CSF samples compared to the concentration needed to counteract the effect of synthetic Aβ42 oligomers in buffer solution. This reduced effectiveness of bapineuzumab in the inhibition of CSF-induced Ca^{2+} influx can have a number of causes including (1) molecular crowding and non-specific binding to a multitude of components present in CSF, which might reduce the effective interaction between the species causing Ca^{2+} influx and the antibody; (2) the morphology of the species present that are responsible for Ca^{2+} influx in CSF might be different from the species in the experiments with purified Aβ; (3) there might be post-translationally modified species of Aβ present in CSF in which the binding epitope of bapineuzumab is absent or inaccessible to the antibody. A combination of these effects could reduce the binding of the antibody to the aggregates, and as a consequence bapineuzumab is less effective at reducing the Ca^{2+} influx induced by CSF.

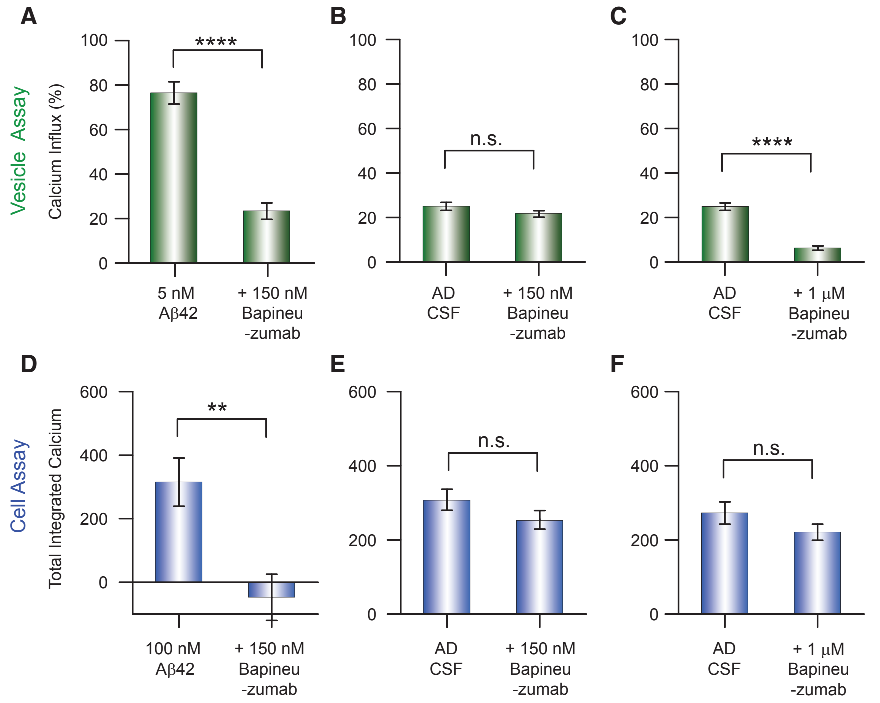

Figure 3. Assessing the Ability of the Antibody Bapineuzumab to Reduce the Ca^{2+} Influx Resulting from the Addition of Aliquots of Human CSF

(A and B) Inhibition of the influx of Ca^{2+} ions into individual vesicles caused by aggregates of recombinant Aβ42 (A) or resulting from the addition of aliquots of AD CSF by bapineuzumab (150 nM) (B).
(C) The inhibition of the influx of Ca^{2+} ions into individual vesicles resulting from the addition of aliquots of CSF by a high concentration of the antibody bapineuzumab (1 μM).
(D and E) Inhibition of the Ca^{2+} influx into astrocytes caused by aggregates of synthetic Aβ42 (D) or resulting from the addition of aliquots of human CSF by the antibody bapineuzumab (150 nM) (E).
(F) The inhibition of the Ca^{2+} influx resulting from the addition of aliquots of human CSF into astrocytes by a high concentration of bapineuzumab (1 μM). The statistical data for these experiments are summarized in Tables S1 and S2. Error bars, SEM.

DISCUSSION

The mechanisms by which amyloid aggregates cause toxic effects and neuronal cell death remain a topic that is under investigation. One commonly proposed mechanism is that aggregates permeabilize membranes, leading to altered calcium homeostasis and, ultimately, to cell death. However, aggregates can also act by a range of other mechanisms such as by causing oxidative stress, neuroinflammation, or synaptic dysfunction. It is therefore extremely important to develop and apply techniques that explore the significance of particular molecular processes that are likely to contribute to the development of AD in humans.

The experiments described above show that human CSF can induce Ca^{2+} influx in our assays using both single vesicles and astrocytes. In addition, our results show that Nb3 and bapineuzumab, both of which were designed to bind to the Aβ peptide, can both inhibit this Ca^{2+} influx. Interestingly, we detected no significant difference in the number of ThT-active species (an indication of β-sheet containing aggregates) or between Ca^{2+} influx induced by aliquots of AD CSF and HC CSF. These results are in agreement with the results of ELISA measurements, using the same antibody to capture and detect aggregates (Yang et al., 2015). This result suggests that the total number of the aggregates by itself is not the critical factor in the onset of AD, but that other factors such as the nature of the aggregates and their effects are also important.

Our results, however, suggest that the other components present in CSF interact with clusterin, Nb3 and bapineuzumab, making them less available to bind the oligomers, or to compete for binding to the aggregates or that the oligomers present are different in their structure or composition from the synthetic Aβ42 oligomers. This means that the levels of clusterin in CSF are not sufficiently high to suppress fully the Ca^{2+} influx. Higher concentrations of bapineuzumab or Nb3 are required to bind to the oligomers and suppress the extent of Ca^{2+} influx caused by CSF compared to the species present in buffer solutions. Bapineuzumab is effective at reducing the Ca^{2+} influx caused by CSF in our vesicle assay, but only at a concentration approximately 100-fold higher than that used in the clinical trial (Goure et al., 2014). If the effects that we observe in CSF reflect those within the brain, this suggests that high concentrations of the antibody (or perhaps higher affinity and more selective antibodies) are required to sequester fully the potentially toxic aggregates. Our *in vitro* assays provide a high-throughput platform to determine if a given antibody is able to reduce the Ca^{2+} influx caused by CSF and the concentration required for it to be effective. This technique can be applied to any therapeutic strategy that targets protein aggregates. It is possible that more effective therapies could be more rapidly developed and optimized if they are tested on human CSF before performing costly clinical trials.

EXPERIMENTAL PROCEDURES

Aβ42 Aggregation

For the cell assays, Hilyte Fluor 647 Aβ42 (Cambridge Bioscience LDT) was aggregated as described previously (Drews et al., 2016).

Aβ Nanobody and Clusterin

Nb3 is an Aβ-specific nanobody isolated from a llama and was prepared as described previously (Drews et al., 2016). Clusterin was obtained as previously described (Drews et al., 2016; Wilson and Easterbrook-Smith, 1992).

Bapineuzumab Equivalent Antibody

The bapineuzumab equivalent antibody was prepared as described in its U.S. patent (US 7179892 B2) in 25 mM histidine, 7% sucrose, and 0.02% polysorbate 80 (pH 6.0) at 48 mg/mL. Endotoxin levels were < 0.005 (EU/mg).

Single Aggregate Visualization through Enhancement Imaging

All CSF samples were imaged with the single aggregate visualization through enhancement (SAVE) method as previously described (Horrocks et al., 2016). In short, a ThT stock solution was prepared in DMSO, diluted into PBS, and filtered (0.02-μm filter, Whatman) with the stock solution prepared daily. Borosilicate glass coverslips were cleaned in an argon plasma cleaner (PDC-002, Harrick Plasma) and coated with poly-(L)-lysine (PLL) for at least 1 hr. The PLL-coated surfaces were washed with PBS before the sample was applied. CSF samples were diluted 10-fold into PBS with a final concentration of 5 μM ThT. Each sample was incubated on the coverslip for 10 min prior to imaging to ensure fixation of the species on the surface. The samples were imaged using a home-built total internal reflection fluorescence (TIRF) microscope. ThT was excited with a 405-nm laser (Oxxius LaserBoxx, LBX-405-100-CIR-PP) aligned to the optical axis of a 1.49 NA TIRF objective (APON60XO TIRF, Olympus, product number N2709400). Imaging was performed in TIRF mode on an inverted Olympus IX-71 microscope with an automated stage (Prior Scientific). The fluorescence signal was recorded on an EMCCD camera (Evolve 512, Photometrics) operating in frame transfer mode (EMGain of 11.5 e−/ADU and 250 ADU/photon) after being separated from the excitation light by a dichroic (Di01-405/488/532/635, Semrock) and a filter (BLP01-488R-25, Semrock). Each pixel was 206 nm in length. For each dataset, 3 × 3 image grids were measured from three different areas of the coverslip with set grid distances to prevent user bias. Images were recorded at 50-ms exposure and 100 frames each field of view in the blue channel (ThT emission). Data analysis was performed as previously described (Horrocks et al., 2016) using ImageJ software, averaging all 100 frames and using the "Find Maxima." The noise tolerance for all measurements was set to 1,000 fluorescent counts. The number of total events was then divided by the image area to give the average number of aggregates per micrometer squared.

CSF Samples

Control CSF samples were collected by lumbar puncture from 6 cognitively normal individuals (aged 49–68 years) and 6 individuals with an AD diagnosis (aged 51–68 years). A standardized protocol for the collection and storage of CSF was followed. In short, lumbar puncture in the L3/L4 interspace was performed between 9 a.m. and 12 a.m. to collect 15 mL of CSF in sterile polypropylene tubes. The samples were de-identified, spun at 3,000 rpm for 10 min, and divided into aliquots each containing 1 mL that were frozen on dry ice and stored at −80°C in 1.5 mL capacity LoBind micro-centrifuge tubes (Eppendorf, Germany). Sample collection, centrifugation, and freezing was completed within 1 hr. CSF $A\beta_{1-42}$, T-tau, and P-tau_{181} were quantified with sandwich ELISAs (INNOTEST β-$amyloid_{1-42}$, hTAU-Ag; Fujirebio Europe, Belgium). Intra-assay coefficients of variation were below 10%. All controls had no cognitive symptoms and a normal CSF T-Tau/$A\beta_{1-42}$ ratio < 0.52. Patients with AD had CSF $A\beta_{1-42}$ < 600 ng/L and T-tau > 350 ng/L. Study protocols were approved by the Queen Square ethics committee (references 12_LO_1504 & 12_LO_005), and all individuals gave written informed consent.

The AD CSF used for the cell assays was collected by lumbar puncture from patients who sought medical advice because of memory problems. The samples were de-identified and aliquoted into 0.5 mL aliquots in polypropylene cryo tubes following centrifugation at 2,200 × *g* and stored at −80°C pending experimental use. CSF $A\beta_{1-42}$, T-tau and P-tau_{181} were quantified with sandwich ELISAs (INNOTEST β-$amyloid_{1-42}$, hTAU-Ag and Phospho-Tau [181P], respectively). All measurements were performed in one round of analyses using one batch of reagents by board-certified laboratory technicians. Intra-assay coefficients of variation were below 10%. All AD positive samples had protein levels of $A\beta_{1-42}$ < 600 ng/L, T-tau > 350 ng/L and P-tau_{181} > 80 ng/L according to Toledo et al. (2015). The study protocol was approved by the regional ethics committee at the University of Gothenburg.

Astrocyte Dosing Experiments

Astrocyte dosing experiments were performed as described previously (Drews et al., 2016) using rat mixed glial preparations. CSF was used undiluted for all dosing experiments. When pre-incubated with clusterin or an anti-/nanobody, the incubation time was 15–30 min before starting experiments, and concentrated solutions of reagents were used so there was negligible, less than 2% dilution for all experiments apart from the 1,000 nM bapineuzumab experiment, where there was 10% dilution.

Single Vesicle Assay

Preparation of Cal-520-Filled Vesicles. Vesicles with a mean diameter of 200 nm were prepared by freeze-and-thaw cycles combined with extrusion as previously described (Flagmeier et al., 2017). Briefly, chloroform stocks of 16:0-18:1 PC (10 mg/mL) and 18:1-12:0 biotin PC (1 mg/mL) were mixed such that the ratio of lipid to biotinylated lipid was 100:1 before the chloroform was removed under vacuum overnight. The lipid mixture was then hydrated in HEPES buffer (50 mM [pH 6.5]) with 100 μM Cal-520. Five freeze-and-thaw cycles were performed using a water bath and dry ice. The lipid solution was extruded 10 times through a membrane with a size cutoff of 200 nm. Size-exclusion chromatography was performed using a Superdex 200 to remove free dye from the surrounding solution. Finally, the size of the vesicles was determined using a Zetasizer (Zetasizer Nano ZSP, Malvern Instruments).

Vesicle Immobilization on a PEGylated Glass Surface. Glass coverslips were cleaned by sonicating in 2% (v/v) Hellmanex III in water for 10 min followed by sonicating twice in water and twice in methanol for 10 min each (Flagmeier et al., 2017). Then the coverslips were dried under a nitrogen stream for 5 min and plasma cleaned in an argon plasma cleaner (PDC-002, Harrick Plasma) for 30 min. The sample chambers were made by affixing Frame-Seal incubation chambers onto the glass slides. For homogeneous surface treatment, 50 μL of a mixture of 100:1 PLL-g-PEG and PLL-g-PEG biotin (both 1 g/L) in HEPES buffer (50 mM, pH 6.5) was added to the coverslip inside of the chamber and incubated for 30 min. The surface was washed three times with filtered HEPES buffer. Then, a solution of NeutrAvidin (50 μL of 0.1 mg/mL in MilliQ) was added and incubated for 15 min, and washed 3 times. Finally, 50 μL of the solution containing vesicles was added to the coverslip and incubated for 30 min before washing carefully at least 5 times with reaction buffer.

Total Internal Reflection Fluorescence Microscope for Single Vesicle Experiment. The measurements of Ca^{2+} influx into individual vesicles was performed using a home-built total internal reflection fluorescence microscope (TIRFM) based on an inverted Olympus IX-71 microscope. A 488-nm laser (Toptica, iBeam smart, 200 mW, Germany) beam was expanded and focused on the back focal plane of the 60×, 1.49 NA oil-immersion objective lens (APON60XO TIRF, Olympus, N2709400) to excite the Cal-520 dye incorporated into the vesicle. The emerging fluorescence was collected by the same objective. The emission beam was separated from the excitation beam by a dichroic (Di01-R405/488/561/635, Semrock), passed through a set of filters (BLP01-488R, Semrock and FF01-520/44-25, Semrock), and imaged onto an air-cooled EMCCD camera (Photometrics Evolve, EVO-512-M-FW- 16-AC-110). Excitation power density was fixed to ~10 W/cm^2 for 50 frames with a scan speed of 20 Hz and bit depth of 16 bits.

Measurement of the Change in Fluorescence Intensity in Individual Vesicles. Hundreds of single vesicles tethered to coverslips via biotin-neutrAvidin linkage were incubated with 50 μL of HEPES buffer (50 mM [pH 6.5]) and imaged as previously described (Flagmeier et al., 2017). Just before the imaging, the HEPES buffer was replaced with 50 μL Ca^{2+} containing buffer solution Leibovitz's L-15 (L15). 16 different fields of view were recorded under three different conditions (background; in the presence of CSF or a solution containing recombinant protein aggregates; and after the addition of ionomycin, respectively). The distance between each field of view was set to 100 μm and was automated (bean-shell script, Micromanager) to avoid any user bias. First, images were acquired in the presence of only L15 buffer, which we denote as blank (F_{blank}). For each field of view, 50 images were taken with an exposure time of 50 ms. Thereafter, a solution of a sample was added and incubated for 10 min before images of the exact same fields of view were recorded (F_{sample}). Next, 10 μL of a solution of ionomycin was added to the coverslip and incubated for 5 min, and, subsequently, images of the vesicles were acquired in the same fields of view.

Data Analysis and Quantification of the Extent of Ca^{2+} Influx. The recorded images were analyzed using Fiji to determine the fluorescence intensity of each spot under the three different conditions, namely background (F_{blank}), in the presence of the sample (F_{sample}), and after the addition of ionomycin ($F_{Ionomycin}$). The relative influx of Ca^{2+} into an individual vesicle due to aggregates of the Aβ42 peptide was then determined using the following equation:

$$Ca^{2+}\text{ influx} = \frac{F_{sample} - F_{blank}}{F_{Ionomycin} - F_{blank}}. \quad \text{(Equation 1)}$$

Single Vesicle Assay Using CSF. For our single vesicle analysis, we took 15 μL of CSF and diluted it two times in the coverslip with 15 μL L15 buffer and incubated for 10 min. Then the change in fluorescence intensity was measured as described above.

Single Vesicle Assay Using CSF with Antibodies or Nanobodies. Indicated concentrations of nanobodies and antibodies were added to samples of CSF and incubated for 15 min before we performed measurements of the change in fluorescence intensity in single vesicles as described above. The dilutions were performed in a manner such that the CSF sample incubated with the vesicles was diluted by a factor of two to allow direct comparisons of the measurements in the absence and presence of antibodies. Comparisons between the samples were made with a two-sample t test using Origin9.

Preparation and Purification of Recombinant Aβ42. The recombinant Aβ42 (M1-42) peptide (MDAEFRHDSGYEVHHQKLVFFAEDVGSNKGAIIGLMVGG VVIA), here called Aβ42, was used for the vesicle assay experiments and prepared as describe previously (Flagmeier et al., 2017; Hellstrand et al., 2010). Briefly, the purification involved sonication of the cells, ion exchange chromatography, and size-exclusion chromatography. Purified samples were aliquoted, lyophilized, and stored at −80°C. For each experiment, solutions of monomeric recombinant Aβ42 were prepared by dissolving the lyophilized Aβ42 peptide in 6 M GuHCl and then purifying the protein using a Superdex 75 10/300 GL. The center of the elution peak was collected, and the peptide concentration was determined from the absorbance of the integrated peak area using ε_{280} = 1,490 L mol^{-1}cm^{-1}.

Aggregation Conditions for Recombinant Aβ42. Aliquots of monomeric Aβ42 were diluted with buffer to a concentration of 2 μM in low-binding Eppendorf tubes on ice before individual samples were then pipetted into multiple wells of a 96-well half-area plate. Then the plate was incubated under quiescent conditions at 37°C, and aliquots for measurements of Ca^{2+} influx were taken at a time point corresponding to the end of the lag phase (approximately 70 min) as previously described (Flagmeier et al., 2017).

Measurement of the Ca^{2+} Influx Caused by Aβ42. Aliquots were taken from an aggregation reaction of recombinant Aβ42 corresponding to the end of the lag phase. To measure the change of the fluorescence intensity in the single vesicle assay, we diluted the sample to 5 nM with L15 buffer as described previously.

Single Vesicle Assay Using Aβ42 with Antibodies or Nanobodies. Indicated concentrations of nanobodies and antibodies were added to an aliquot taken from an aggregation reaction of recombinant Aβ42 and incubated for 15 min. The change in the fluorescence intensity was measured as described above.

SUPPLEMENTAL INFORMATION

Supplemental Information includes three figures and three tables and can be found with this article online at https://doi.org/10.1016/j.celrep.2017.11.057.

ACKNOWLEDGMENTS

We are grateful to Dr. Damian Crowther, the director of Neuroscience, Innovative Medicines and Early Development, AstraZeneca for critical comments. S.D. is funded by a Marie Skłodowska-Curie Individual Fellowship (701013). P.F. received funding through PhD fellowships from the Boehringer Ingelheim Fonds and the Studienstiftung des deutschen Volkes. D.C.W. is funded by the EPSRC. D.R.W. was funded by Alzheimer's research UK (ARUK-PPG-2015A-1). P.F. and C.M.D. thank the Cambridge Centre for Misfolding Diseases for support. C.M.D. is supported by the Wellcome Trust and the UK Biotechnology and Biological Sciences Research Council. H.Z. is a Wallenberg Academy Fellow and is additionally funded by the Swedish Research Council (2013-2546) and the European Research Council (681712). S.G. is funded by the Wellcome Trust (100172) and the NIHR Biomedical Research Centre UCLH. Funding for the CSF sample collections came from the Leonard Wolfson Experimental Neurology Centre, Alzheimer's Research UK, NIHR Queen Square Dementia Biomedical Research Unit, and University College London Hospitals Biomedical Research Centre. D.K. is a Royal Society Glaxo Wellcome Professor of Molecular Medicine and funded by the European Research Council (669237).

AUTHOR CONTRIBUTIONS

A.D. and W.-H.C. performed the cellular assay; S.D. and P.F. carried out the single vesicle assay; D.C.W. and D.R.W. performed the SAVE imaging; and D.C.W. and M.R. carried out immuno-depletion. C.V. and S.M. provided the Nb3 nanobody, and R.W.P., C.F.S., N.C.F., J.M.S., H.Z., and S.G. provided the CSF samples. D.K. conceived and supervised the project with the help of C.M.D. All the authors wrote the manuscript.

DECLARATION OF INTERESTS

The authors declare no competing interests.

Received: June 30, 2017
Revised: September 28, 2017
Accepted: November 15, 2017
Published: December 12, 2017

REFERENCES

Arbel-Ornath, M., Hudry, E., Boivin, J.R., Hashimoto, T., Takeda, S., Kuchibhotla, K.V., Hou, S., Lattarulo, C.R., Belcher, A.M., Shakerdge, N., et al. (2017). Soluble oligomeric amyloid-β induces calcium dyshomeostasis that precedes synapse loss in the living mouse brain. Mol. Neurodegener. *12*, 27.

Benilova, I., Karran, E., and De Strooper, B. (2012). The toxic Aβ oligomer and Alzheimer's disease: an emperor in need of clothes. Nat. Neurosci. *15*, 349–357.

Blennow, K., Zetterberg, H., Rinne, J.O., Salloway, S., Wei, J., Black, R., Grundman, M., and Liu, E.; AAB-001 201/202 Investigators (2012). Effect of immunotherapy with bapineuzumab on cerebrospinal fluid biomarker levels in patients with mild to moderate Alzheimer disease. Arch. Neurol. *69*, 1002–1010.

Chiti, F., and Dobson, C.M. (2017). Protein misfolding, amyloid formation, and human disease: a summary of progress over the last decade. Annu. Rev. Biochem. *86*, 27–68.

Drews, A., Flint, J., Shivji, N., Jönsson, P., Wirthensohn, D., De Genst, E., Vincke, C., Muyldermans, S., Dobson, C., and Klenerman, D. (2016). Individual aggregates of amyloid beta induce temporary calcium influx through the cell membrane of neuronal cells. Sci. Rep. *6*, 31910.

Flagmeier, P., De, S., Wirthensohn, D.C., Lee, S.F., Vincke, C., Muyldermans, S., Knowles, T.P.J., Gandhi, S., Dobson, C.M., and Klenerman, D. (2017). Ultrasensitive measurement of Ca(2+) influx into lipid vesicles induced byp aggregates. Angew. Chem. Int. Ed. Engl. *56*, 7750–7754.

Glenner, G.G., and Wong, C.W. (1984). Alzheimer's disease: initial report of the purification and characterization of a novel cerebrovascular amyloid protein. Biochem. Biophys. Res. Commun. *120*, 885–890.

Goure, W.F., Krafft, G.A., Jerecic, J., and Hefti, F. (2014). Targeting the proper amyloid-beta neuronal toxins: a path forward for Alzheimer's disease immunotherapeutics. Alzheimers Res. Ther. *6*, 42.

Haass, C., and Selkoe, D.J. (2007). Soluble protein oligomers in neurodegeneration: lessons from the Alzheimer's amyloid beta-peptide. Nat. Rev. Mol. Cell Biol. *8*, 101–112.

Hellstrand, E., Boland, B., Walsh, D.M., and Linse, S. (2010). Amyloid β-protein aggregation produces highly reproducible kinetic data and occurs by a two-phase process. ACS Chem. Neurosci. *1*, 13–18.

Horrocks, M.H., Lee, S.F., Gandhi, S., Magdalinou, N.K., Chen, S.W., Devine, M.J., Tosatto, L., Kjaergaard, M., Beckwith, J.S., Zetterberg, H., et al. (2016). Single-molecule imaging of individual amyloid protein aggregates in human biofluids. ACS Chem. Neurosci. *7*, 399–406.

Klyubin, I., Betts, V., Welzel, A.T., Blennow, K., Zetterberg, H., Wallin, A., Lemere, C.A., Cullen, W.K., Peng, Y., Wisniewski, T., et al. (2008). Amyloid beta protein dimer-containing human CSF disrupts synaptic plasticity: prevention by systemic passive immunization. J. Neurosci. *28*, 4231–4237.

Liu, E., Schmidt, M.E., Margolin, R., Sperling, R., Koeppe, R., Mason, N.S., Klunk, W.E., Mathis, C.A., Salloway, S., Fox, N.C., et al.; Bapineuzumab 301 and 302 Clinical Trial Investigators (2015). Amyloid-β 11C-PiB-PET imaging results from 2 randomized bapineuzumab phase 3 AD trials. Neurology *85*, 692–700.

Luo, J., Wärmländer, S.K.T.S., Gräslund, A., and Abrahams, J.P. (2016). Cross-interactions between the Alzheimer disease amyloid-β peptide and other amyloid proteins: a further aspect of the amyloid cascade hypothesis. J. Biol. Chem. *291*, 16485–16493.

Miles, L.A., Crespi, G.A.N., Doughty, L., and Parker, M.W. (2013). Bapineuzumab captures the N-terminus of the Alzheimer's disease amyloid-beta peptide in a helical conformation. Sci. Rep. *3*, 1302.

Narayan, P., Orte, A., Clarke, R.W., Bolognesi, B., Hook, S., Ganzinger, K.A., Meehan, S., Wilson, M.R., Dobson, C.M., and Klenerman, D. (2011). The extracellular chaperone clusterin sequesters oligomeric forms of the amyloid-β(1-40) peptide. Nat. Struct. Mol. Biol. *19*, 79–83.

Narayan, P., Meehan, S., Carver, J.A., Wilson, M.R., Dobson, C.M., and Klenerman, D. (2012). Amyloid-β oligomers are sequestered by both intracellular and extracellular chaperones. Biochemistry *51*, 9270–9276.

Narayan, P., Holmström, K.M., Kim, D.H., Whitcomb, D.J., Wilson, M.R., St George-Hyslop, P., Wood, N.W., Dobson, C.M., Cho, K., Abramov, A.Y., and Klenerman, D. (2014). Rare individual amyloid-β oligomers act on astrocytes to initiate neuronal damage. Biochemistry *53*, 2442–2453.

Riek, R., and Eisenberg, D.S. (2016). The activities of amyloids from a structural perspective. Nature *539*, 227–235.

Salloway, S., Sperling, R., Fox, N.C., Blennow, K., Klunk, W., Raskind, M., Sabbagh, M., Honig, L.S., Porsteinsson, A.P., Ferris, S., et al.; Bapineuzumab 301 and 302 Clinical Trial Investigators (2014). Two phase 3 trials of bapineuzumab in mild-to-moderate Alzheimer's disease. N. Engl. J. Med. *370*, 322–333.

Savage, M.J., Kalinina, J., Wolfe, A., Tugusheva, K., Korn, R., Cash-Mason, T., Maxwell, J.W., Hatcher, N.G., Haugabook, S.J., Wu, G., et al. (2014). A sensitive aβ oligomer assay discriminates Alzheimer's and aged control cerebrospinal fluid. J. Neurosci. *34*, 2884–2897.

Shankar, G.M., Li, S., Mehta, T.H., Garcia-Munoz, A., Shepardson, N.E., Smith, I., Brett, F.M., Farrell, M.A., Rowan, M.J., Lemere, C.A., et al. (2008). Amyloid-β protein dimers isolated directly from Alzheimer's brains impair synaptic plasticity and memory. Nat. Med. *14*, 837–842.

Toledo, J.B., Zetterberg, H., van Harten, A.C., Glodzik, L., Martinez-Lage, P., Bocchio-Chiavetto, L., Rami, L., Hansson, O., Sperling, R., Engelborghs, S., et al.; Alzheimer's Disease Neuroimaging Initiative (2015). Alzheimer's disease cerebrospinal fluid biomarker in cognitively normal subjects. Brain *138*, 2701–2715.

Vandenberghe, R., Rinne, J.O., Boada, M., Katayama, S., Scheltens, P., Vellas, B., Tuchman, M., Gass, A., Fiebach, J.B., Hill, D., et al.; Bapineuzumab 3000 and 3001 Clinical Study Investigators (2016). Bapineuzumab for mild to moderate Alzheimer's disease in two global, randomized, phase 3 trials. Alzheimers Res. Ther. *8*, 18.

Vranová, H.P., Hényková, E., Kaiserová, M., Menšíková, K., Vaštík, M., Mareš, J., Hluštík, P., Zapletalová, J., Strnad, M., Stejskal, D., and Kaňovský, P. (2014). Tau protein, beta-amyloid$_{1-42}$ and clusterin CSF levels in the differential diagnosis of Parkinsonian syndrome with dementia. J. Neurol. Sci. *343*, 120–124.

Walsh, D.M., Klyubin, I., Fadeeva, J.V., Cullen, W.K., Anwyl, R., Wolfe, M.S., Rowan, M.J., and Selkoe, D.J. (2002). Naturally secreted oligomers of amyloid β protein potently inhibit hippocampal long-term potentiation in vivo. Nature *416*, 535–539.

Wilson, M.R., and Easterbrook-Smith, S.B. (1992). Clusterin binds by a multivalent mechanism to the Fc and Fab regions of IgG. Biochim. Biophys. Acta *1159*, 319–326.

Yang, T., O'Malley, T.T., Kanmert, D., Jerecic, J., Zieske, L.R., Zetterberg, H., Hyman, B.T., Walsh, D.M., and Selkoe, D.J. (2015). A highly sensitive novel immunoassay specifically detects low levels of soluble Aβ oligomers in human cerebrospinal fluid. Alzheimers Res. Ther. *7*, 14.

Yerbury, J.J., and Wilson, M.R. (2010). Extracellular chaperones modulate the effects of Alzheimer's patient cerebrospinal fluid on Abeta(1-42) toxicity and uptake. Cell Stress Chaperones *15*, 115–121.

Yerbury, J.J., Poon, S., Meehan, S., Thompson, B., Kumita, J.R., Dobson, C.M., and Wilson, M.R. (2007). The extracellular chaperone clusterin influences amyloid formation and toxicity by interacting with prefibrillar structures. FASEB J. *21*, 2312–2322.

Cell Reports, Volume *21*

Supplemental Information

Inhibiting the Ca^{2+} Influx Induced by Human CSF

Anna Drews, Suman De, Patrick Flagmeier, David C. Wirthensohn, Wei-Hsin Chen, Daniel R. Whiten, Margarida Rodrigues, Cécile Vincke, Serge Muyldermans, Ross W. Paterson, Catherine F. Slattery, Nick C. Fox, Jonathan M. Schott, Henrik Zetterberg, Christopher M. Dobson, Sonia Gandhi, and David Klenerman

Supplemental Figures:

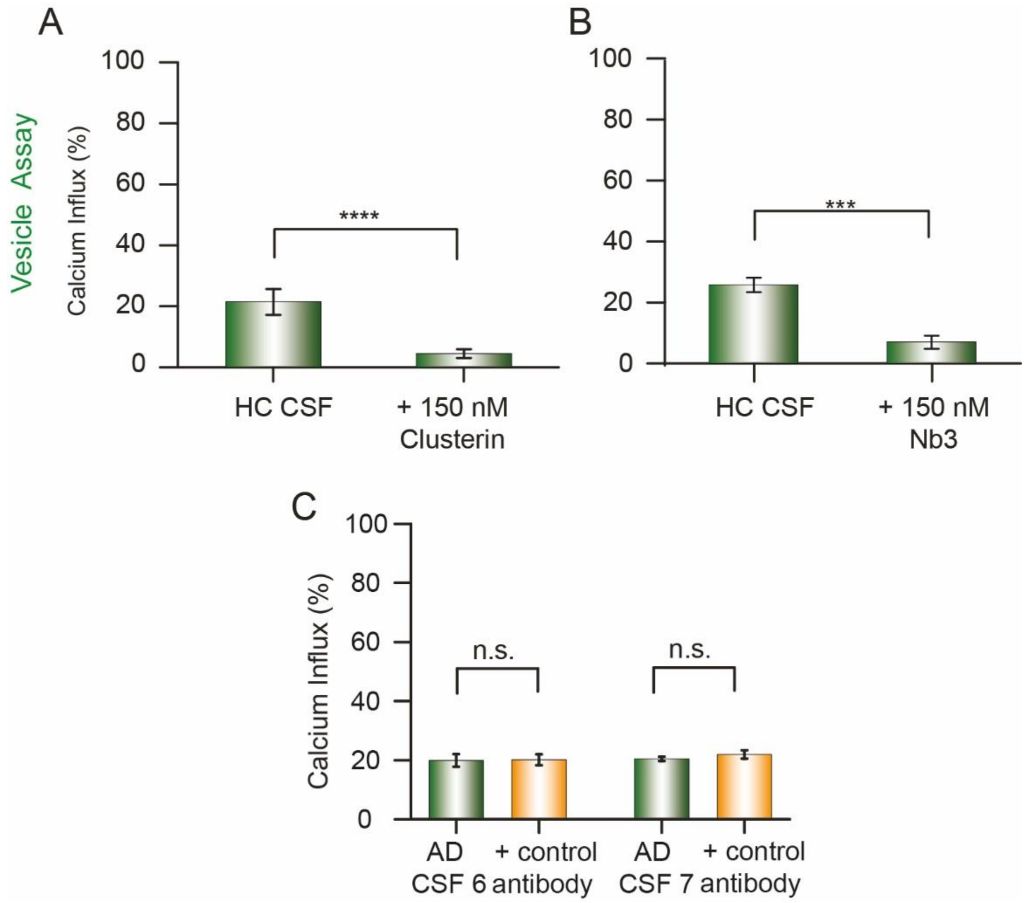

Figure S1. Assessing the ability of Clusterin and Nb3 to counteract the calcium influx caused by human HC CSF and effect of control anti GFP antibody on CSF induced Calcium influx. Related to Figure 2. Inhibition of the calcium influx caused by HC CSF with Clusterin **(A)** and Nb3 **(B)** ,the error bars represent SEM. The statistical data for the experiments are summarized in Table S1. **(C)** A control antibody, 1 μM anti GFP antibody, does not show any significant change in CSF induced Calcium influx.

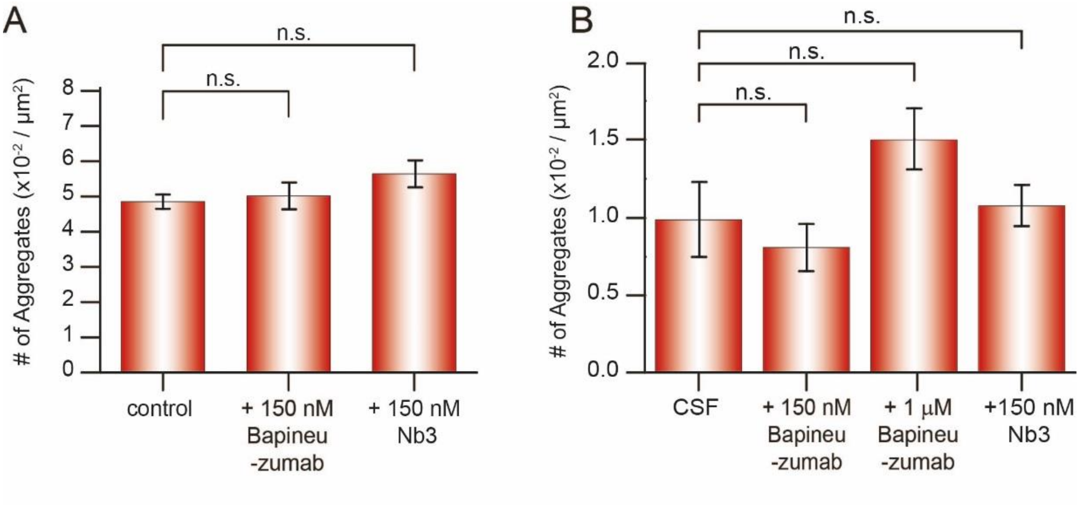

Figure S2. Effect of Antibodies on Thioflavin T positive Aβ42 aggregates and human CSF. **Related to Figure 3. (A)** Aβ42 was aggregated at 5 µM for 5h at 37° C in PBS. An aggregated solution of 1 µM total Aβ4*2* monomer concentration was incubated with 150 nM of each antibody for 15 min and aggregates were counted as described previously. Each bar represents the mean of 3 slides with 27 field of views overall imaging a 3 x 10^6 μm^2 area. Error bars represent the standard error of the mean of all 27 field of views **(B)** The effect of the antibodies on the number of ThT positive aggregates in human CSF. The mean of 4 samples with 27 field of views each with the error bars representing the standard error of the mean of all samples. Full statistical details are found in Table S3.

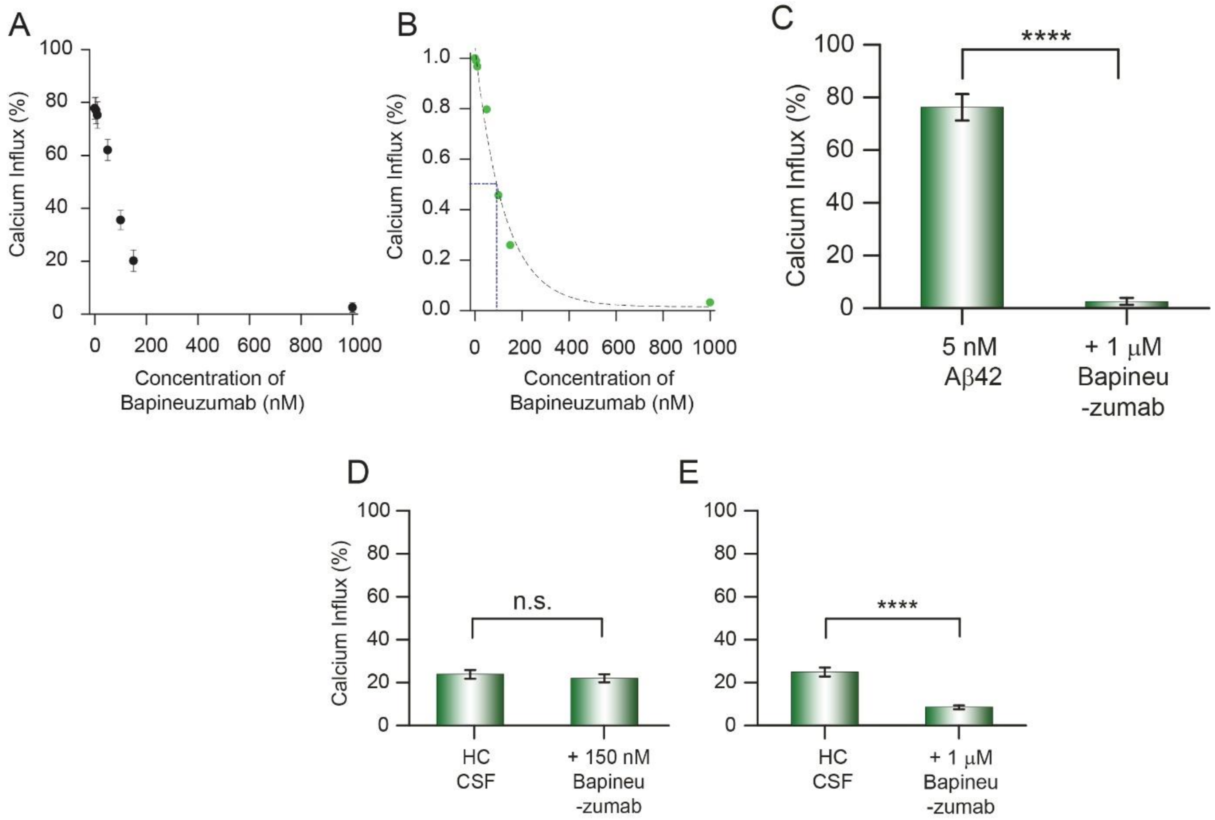

Figure S3. Inhibition of CSF induced Calcium influx by Bapineuzumab. Related to Figure 3. Recombinant Aβ42 was incubated at a concentration of 2 μM at 37 °C and an aliquot was taken from the reaction at a time point corresponding to the end of the lag-phase [1]. **(A)** The aliquot was diluted to a concentration of 5 nM and incubated with increasing concentrations of Bapineuzumab. **(B)** The Ca^{2+} influx normalized to the influx in the absence of Bapineuzumab. The Ca^{2+} influx caused by the Aβ42 aggregates was reduced by half at a concentration of approximately 90 nM of Bapineuzumab (indicated by the blue line). Lines are guides to the eye. **(C)** Inhibition of the calcium influx caused by recombinant Aβ42 aggregates by the antibody Bapineuzumab at a concentration of 1 μM measured with the vesicle assay. Error bars are SEM. **(D)** and **(E)** Inhibition of the calcium influx caused by human HC CSF into vesicles by 150 nM and 1μM Bapineuzumab respectively. The error bars are SEM.

Supplemental Tables:

Table S1: Statistical data to all vesicle assays Related to Figure 1, 2 and 3.

Single Vesicle Assay				
**Figure No.**	**Sample**	**Total vesicle analysed**	**No of CSF samples**	**P Value**
**1D**	***AD***	4906	5	0.440500
	HC	4602	5	
**2A**	***AD***	5032	5	0.000033
	AD+ Clusterin	4876	5	
**Fig. S1A**	***HC***	4905	5	0.000040
	HC+ Clusterin	5536	5	
**2B**	***AD***	4690	5	0.000016
	AD+ Nb3	4883	5	
**Fig. S1B**	***HC***	4925	5	0.000110
	HC + Nb3	4754	5	
**3A**	***Aβ42***	5826	5	0.000034
	Aβ42+ 150nM BAPI	4869	5	
**3B**	***AD***	5429	5	0.18411
	AD+ 150 nM BAPI	5202	5	
**3C**	***AD***	5277	5	0.00112
	AD+ 1 µm BAPI	5509	5	

Table S2: Statistical data to all cell assays. Related to Figure 1, 2 and 3.

Cell Assay				
**Figure No.**	**Sample**	**No of Cells used**	**No of CSF samples**	**P Value**
**1E**	***AD***	33	6	0.306118
	HC	65	9	
**2C**	***AD***	25	4	0.001751
	AD+ Clusterin	19	4	
**2D**	***AD***	33	4	0.000591
	AD+ Nb3	24	4	
**3D**	***Aβ42***	8	1	0.004945
	Aβ42 + BAPI	6	1	
**3E**	***AD***	37	4	0.279328
	AD + 150 nM BAPI	25	4	
**3F**	***AD***	31	5	0.166927
	AD + 1000 nM BAPI	27	5	

Table S3: Statistical data to all SAVE measurements. Related to Figure 1.

Single Aggregate Measurements				
**Figure No.**	**Sample**	**Areas imaged per sample (overall 3 x 10^6 μm^2)**	**No of CSF samples**	**P Value**
**1F**	***AD***	27	19	0.4907
	HC	27	12	
**S3A**	***Aβ42***	27	N/A	0.703221
	Aβ42 + BAPI	27	N/A	
**S3A**	***Aβ42***	27	N/A	2.05552
	Aβ42 + Nb3	27	N/A	
**S3B**	***CSF***	27	4	0.626095
	CSF + 150 nM BAPI	27	4	
**S3B**	***CSF***	27	4	0.289404
	CSF + 1000 nM BAPI	27	4	
**S3B**	***CSF***	27	4	0.730615
	CSF + 150 nM Nb3	27	4	
